# Strontium/copper doped nano-bioactive glass for biomedical applications

**DOI:** 10.1038/s41598-025-27551-3

**Published:** 2025-11-27

**Authors:** Manar M. Ahmed, Amany A. El-Kheshen, Mona Moaness

**Affiliations:** 1 Glass Research Department, Advanced Materials Technology and Mineral Resources Research Institute, National Research Centre, 33 El Bohouth St., Dokki, PO Box 12622, Cairo, Egypt; 2 Refractories, Ceramics and Building Materials Department, Advanced Materials Technology and Mineral Resources Research Institute, National Research Centre, 33 El Bohouth St., Dokki, PO Box 12622, Cairo, Egypt

**Keywords:** Bioactive glass, Copper/Strontium doping, Biocompatibility, Controlled drug delivery, Antimicrobial effect, Cytotoxicity, Biophysics, Materials science, Nanoscience and technology

## Abstract

This work is reported for the preparation and characterization of nano bioactive glass doped with copper and strontium in the system (SiO_2_-CaO-P_2_O_5_-CuO-SrO).Compared to traditional procedures, the Sol-gel approach offers a number of advantages for glass formation, including improved control over size and morphology. At the expense of CaO, CuO and SrO were added to the glass compositions. CuO and SrO were used for their highly antibacterial effects and bioactivity enhancement. To confirm the bioactivity of all the glass samples, scanning electron microscopy (SEM), Brunauer Emmett Teller (BET) surface area measurements, Fourier transform infrared spectroscopy (FTIR), and X-ray diffraction (XRD) were used to assess the morphological and structural characteristics of the produced glasses. The particles that developed on the glass samples’ surface during immersion in the simulated body fluid (SBF) were analysed to detect the glasses’ bioactivity. The hydroxyapatite (HAp) layer’s development was verified by EDX analysis. ICP (Inductively Coupled Plasma) Spectroscopy was used to analyse the ion release in the SBF solution. Additionally, the impact of the antibacterial activity on several types of bacteria (including *Gram-positive*,* Gram-negative*,* and antifungal bacteria*) was examined. Moreover, Ciprofloxacin was used as a model medication to test the drug loading efficiency. The Korsmeyer-Peppas kinetic model and zero order models were used to study the drug release mechanisms. The cytotoxicity of the nano-bioactive glass against human bone osteosarcoma cells (MG-63) and bone marrow stromal cells was also evaluated using the MTT assay.

## Introduction

Bioactive glasses (BGs) are now well-established biomaterials used in orthopaedics and bone tissue regeneration. The first scientific evidence of bioactive glasses’ potential to bind with bones dates back to the second half of the twentieth century, due to the work of Prof. Hench and colleagues^1,2^. It became the “gold standard” of what eventually developed the family of bioactive glasses, mostly because of its osteoinductive and osteoconductive qualities^3^.The constituents of glass, including elements such as Si, Ca, Na, P, Sr, and Cu, significantly influence the material’s properties and contribute to various biological behaviors both in vitro and in vivo^4^. ]. Among these, silicon is a major component of silicate glasses and typically exists as the silicate ion (Si⁴⁺) in biological environments. Silicon plays a vital role in metabolic processes involved in bone formation. Previous studies have identified silicon as an essential element in glycols-amino-glycan and their associated protein complexes, which are plentiful in bone and connective tissues, suggesting its importance in bone growth and maintenance^5^. During the initial stages of bio-mineralization at sites of active calcification, silicon levels were found to rise concurrently with calcium at low calcium concentrations. However, silicon concentration tends to decline as the mineral phase approaches the composition of hydroxyapatite (HA). These observations support the hypothesis that early bone calcification involves elevated silicon levels, which may facilitate HA precipitation within the matrix^6^. Furthermore, in vitro studies have demonstrated that the silicate ion promotes osteoblast differentiation and proliferation in a dose-dependent manner and enhances collagen type 1 production^7^. The central silicon atom can form two distinct types of electronic configurations with oxygen atoms, known as bridging oxygen (BO) and non-bridging oxygen (NBO). Bridging oxygen occurs when an oxygen atom shares its two unpaired electrons with two adjacent silicon atoms, creating covalent bonds with both. In the formation of non-bridging oxygen (NBO), each oxygen atom donates one unpaired electron to form a covalent bond with a neighboring silicon atom, while the second unpaired electron engages in an ionic interaction with alkali or alkaline earth metal ions—commonly known as network modifiers (e.g., Ca²⁺, Na⁺, Sr²⁺, Cu²⁺)^8^. Variations in the concentration of NBOs caused by the incorporation of such modifiers lead to disruptions in the continuity of the glass network. As a result, the physical, chemical, thermal, and solubility properties of the glass can be significantly altered^9^. When bio glasses are implanted into living tissue, they can demonstrate various bioactive responses. These include the formation of a hydroxycarbonate apatite layer on their surface, enabling chemical bonding with bone. Additionally, bio glasses can stimulate stem cell behavior—such as migration and differentiation—as well as promote bone cell activities, including proliferation, adhesion, and migration. They may also influence enzymatic functions and support angiogenesis, depending on their composition and structure^9^. Phosphorus (P) is the second most abundant mineral in the human body and is found in various organic compounds, including nucleic acids, proteins, phospholipids, carbohydrates, and ATP. It plays a vital role in numerous physiological processes, particularly in skeletal development and remodeling. Research has shown that phosphorus is essential for the formation of secondary ossification centers, maturation of the growth plate, mineralization of the extracellular matrix, and osteoblast differentiation^8,10^. Calcium (Ca), a key component of silicate glasses, plays multiple roles in biological systems, ranging from providing structural support at the macroscopic level to participating in intracellular signaling processes^4^. In vitro studies have shown that moderate extracellular calcium concentrations are optimal for promoting extracellular matrix (ECM) mineralization and osteoblast proliferation. Additionally, research has demonstrated that incorporating calcium into scaffolds enhances the adhesion, proliferation, and differentiation of osteoblast-like MG63 cells. These findings highlight the potential of Ca ions in promoting bone regeneration for biomedical applications, emphasizing calcium’s fundamental role in bone formation and cellular signaling^11^. The suitability of glass materials for implantation in living organisms largely depends on their solubility characteristics. In particular, the solubility or degradability of silica-based glasses can be tailored by adjusting their silica content. Bioactivity is generally linked to the glass dissolution process^12^. Typically, the solubility of these glasses decreases as the silica content increases, unless specific ions are introduced to alter their dissolution behavior^12^. Among the different morphologies of bioactive glasses (BGs), bioactive glass nanoparticles (BGNs) have gained significant attention for a wide range of biomedical applications. This is primarily due to their nanoscale size, high specific surface area, and large surface-to-volume ratio, which impart unique properties. These morphological features give BGNs a distinct advantage over their micron-sized counterparts in certain applications, such as drug delivery^13^. The sol-gel technique has become increasingly favored in recent decades due to its advantages, including high purity and low-temperature processing. Specifically, this method can be conducted at room temperature, which helps prevent the evaporation of volatile precursors such as P₂O₅. As a result, the process yields a final product with enhanced homogeneity and purity. Sol gel route for glass synthesis is favored for preparing Nano- sized glass material^14^.

Researchers are focusing on including elements such as Zn, Mg, Ag, Cu and Si into the composition of these bioactive glasses to improve their physical and biological efficiency^15^. Notably, copper and strontium ions have piqued the interest of researchers since they have been shown to give osteogenesis, angiogenesis, and antimicrobial capabilities to bioactive glass. Cu^2+^ ions are known to boost endothelial cell proliferation, increase osteoblastic cell activity and proliferation, improve micro-vessel creation, aid wound healing, and have antimicrobial properties. Furthermore, copper ions are not harmed during the high-temperature manufacturing of scaffolds. It has been shown that Sr^2+^ ions preserve the exceptional cellular bioactivity of the glasses while simultaneously increasing osteoblast activity and inhibiting osteoclast development. Additionally, it has been found that Sr^2+^ ion release significantly raises alkaline phoaphate activity (ALP). However, because a high Sr content will affect glass network formation and decrease the degree of order in the mesoporous structure, its doping amount inside the glass needs to be carefully controlled^16^.

Bacterial infections can delay wound healing and even result in surgical failure. BGs have previously been mentioned for their antibacterial effects; the kind of *Staphylococcus aureus* that is a major cause of hospital-acquired infections and is antibiotic resistant. Some research has been done to improve the antibacterial efficiency of BGs. It has been proposed, for example, that including Sr^2+^ into the composition of biomaterials limits bacterial growth^17^.

One common antibiotic used to treat bone-infective illnesses, particularly osteomyelitis, is ciprofloxacin. Its broad-spectrum antibacterial qualities against germs linked to osteomyelitis are the reason for this. Furthermore, it has nothing to do with the emergence of a bacterial strain that is resistant^18^. Moreover, ciprofloxacin can enter bone quickly and reach quantities greater than those found in plasma. The physical qualities of ciprofloxacin are linked to its earlier characteristics, which can thus regulate drug release. The three physical characteristics that most influence ciprofloxacin release are solubility, pH, and temperature. Its low solubility restricted its release, and the medium’s pH also affected it; it releases more in basic or acidic solutions than in neutral ones^19–21^.

In this study, glasses containing Sr and Cu (with concentrations varying from 5 to 10 weight%) were synthesized via sol-gel technique. Investigations were conducted into the glasses’ structural qualities as well as the physicochemical and morphological traits of the calcium phosphate layer that formed on their surfaces during soaking in simulated bodily fluid (SBF) solution. Acceptable analytical methods include FTIR, X-ray diffraction (XRD), and scanning electron microscopy (SEM) were utilized .Additionally, the drug loading efficiency was tested using Ciprofloxacin as a model pharmaceutical. The drug release processes were investigated using zero order models and the Korsmeyer-Peppas kinetic model. The MTT assay was also used to assess the Nano-bioactive glass’s cytotoxicity against human bone osteosarcoma cells (MG-63) and bone marrow stromal cells.

## Experimental

### Sol-gel synthesis of bioactive glass nanoparticles

Tetraethyl orthosilicate (TEOS), calcium nitrate tetra hydrate Ca (NO)_3_.4H_2_O, copper nitrate Cu(NO_3_)_2_, strontium nitrate Sr(NO_3_)_2_, and triethyl phosphate (TEP) were among the materials utilized to make the sol-gel generated glass. Fluka (Buchs, Switzerland) supplied all of these materials. Merck supplied the nitric acid (68%) and ammonia solution (33%).

In the SiO_2_-CaO-P_2_O_5_-CuO-SrO system, Prepared Nano bioactive glass compositions. CuO and SrO were substituted for CaO in the glass compositions. Table 1 lists the nominal compositions and codes of the prepared glasses. Initially, (16.89) ml of tetraethyl orthosilicate, (16.17) ml of distilled water, and (2.69) ml of 2 M nitric acid (as a hydrolysis catalyst), were successively mixed in (52.43) ml of ethanol and the resulting mixture was permitted to react for 60 min under continuous magnetic stirring for the acid hydrolysis of TEOS. Subsequently, the precise quantity of (14.75) g of Ca(NO_3_)_2_·4H_2_O was introduced, permitting a 30-minute duration for the reagent to undergo complete reaction. Subsequently, precise quantities of several reagents were introduced in this sequence: (4.76) ml of TEP, followed by (1.03 g and 2.06 g for Sr nitrate at 5% and 10% respectively), and (1.53 g and 3.06 g for Cu nitrate at 5% and 10% respectively). Each reagent was allowed a full 30 min for complete reaction. To ensure the completion of hydrolysis, mixing continued for an extra 60 min following the final addition. Subsequently, the mixture was subjected to a conventional ultrasonic bath operating at a frequency of 50–60 kHz and power ranging from 100 to 200 W. A mechanical stirrer was employed to agitate the mixture vigorously as 20 ml of a 2 M ammonia solution, serving as a gelation catalyst, was introduced. The mixture solidified within a matter of minutes. To prevent the development of a bulk gel, the mixture underwent mechanical stirring throughout the gelation process, complemented by ultrasonic vibration. Finally, the gels that were produced underwent a drying process in an oven for duration of two days at a temperature of 75 °C. The gels underwent stabilization through a heat treatment process, maintained at a consistent rate of 3 °C/min until reaching 550 °C for a duration of 2 h, resulting in the formation of glass powders.

**Table 1:** The nominal compositions and codes of the prepared samples.

Sample code	Composition (wt %)				
	SiO_2_	CaO	*P*_2_O_5_	SrO	CuO
BCuSr 0%	45	35	20	0	0
BCuSr 5%	45	25	20	5	5
BCuSr 10%	45	15	20	10	10

### Characterization of prepared samples

#### Transmission electron microscope (TEM) analysis

The morphology and dimensions of sol-gel CuO and SrO doped bioactive glass nanoparticles were examined utilizing a TEM (JEM2010, Japan) operating at 200 kV. The elemental composition of glasses was assessed using energy dispersive X-ray (EDX) analysis utilizing a JEOL JXA-840 A electron probe micro-analyzer.

#### XRD analysis

The phase analysis of the dried gels, subjected to heating at 550 °C, was conducted using Ni-filtered CuKα irradiation at 40 KV and 25 mA with a BRUKER axs, D8ADVANCE X-ray diffractometer. The samples underwent XRD analysis prior to immersion in the SBF to confirm their amorphous nature.

#### FTIR analysis

The generated glasses’ infrared spectra were obtained using a Fourier Transformer Infrared Spectrophotometer (FT-IR) (model FT/IR-6100 type A). The 400–4000 cm^− 1^ wave number range was used to capture the spectra.

#### Textural analysis of glass nanoparticles

A High-Speed Gas Sorption Analyzer (NOVA 2000 series, Chromatic, UK) was used to measure the nitrogen adsorption-desorption isotherms at 77 K. Prior to measurement, the materials were out-gassed in a vacuum for six hours at 150 °C. The Barrett-Emmett-Teller (BET) method was used to determine the specific surface areas. From the adsorption branches of the isotherms, the pore volume, average pore diameter, and pore-size distributions were detected using the Barrett-Joyner-Halanda (BJH) method. The total pore volume was determined using the amount adsorbed at a maximum relative pressure. USA: Micrometric (19321).

### Assessment of bioactivity

To characterize the hydroxyapatite layer that developed on the surfaces of the prepared samples (BCuSr 0%, BCuSr 5%, and BCuSr 10%), the samples were submerged in a simulated body fluid (SBF), which is an aqueous solution containing a range of inorganic ions at concentrations akin to those found in human blood plasma^22^. This was conducted to assess the bioactive characteristics of the synthesized samples in vitro. Following a two-week immersion in SBF, the discs were extracted and analyzed using a scanning electron microscope paired with energy-dispersive X-ray spectroscopy (JEOL JXA-840 A, Electron probe micro-analyzer, Japan) at 15KV. This facilitated the analysis of the glass’s morphology, porosity, and elemental constituents.

Discs of the examined samples were immersed in simulated body fluid for different durations (3 h, 1 day, 2 days, 4 days, 7 days, and 14 days) and maintained at a temperature of 37 °C with a pH of 7.4 during incubation. Inductively coupled plasma and spectrophotometric analyses were employed to characterize the ionic constituents released from samples into simulated body fluid at designated intervals.

### Drug loading into the bioactive glass

Ciprofloxacin was utilized in this study for the purpose of conducting drug release experiments. Ciprofloxacin is a pharmaceutical compound utilized for the treatment of various bacterial infections. Consequently, it was utilized as a representative compound in this study. A concentration of 1 mg/mL of ciprofloxacin solution was employed. The bioactive glass nanoparticles underwent immersion in 8 ml of drug solution at a controlled temperature of 37 °C for duration of two days, followed by a drying process that lasted four days. Subsequently, the drug solution was extracted. The variation in drug concentration prior to and following sample immersion was utilized to determine drug uptake by glass nanoparticles. The concentrations of ciprofloxacin were quantified utilizing a UV spectrophotometer calibrated to a wavelength of 277 nm.

### Determination of the cumulative drug released from the bioactive glass

Each glass sample containing medication was submerged in a 10 ml Tris buffer solution to assess the concentration of ciprofloxacin released from the bioactive glass nanoparticles into the Tris buffer. Following several time intervals, an extraction of 3 ml from this solution was performed, subsequently substituting it with an equivalent volume of fresh 3 ml Tris buffer solution. For the purpose of concentration assessments, the eluted samples were subjected to freezing at -4 °C. A UV spectrophotometer calibrated to 277 nm was employed to quantify the concentration of ciprofloxacin released in vitro. Additionally, reference solutions with established drug concentrations were prepared and assessed using the identical methodology as the samples to determine the unknown drug concentrations.

The dissolution data were analyzed using the Korsmeyer-Peppas and zero-order models, leading to the identification of the drug release mechanism within the Korsmeyer-Peppas framework^23^. This model, designed to establish a direct correlation for identifying these mechanisms, graphs log (drug release concentration %) versus log time utilizing Eq. (1) to elucidate the mechanism of drug release from the glass nanoparticles system.

1 $${{\text{Q}}_{\text{t}}}={\text{A}}{{\text{t}}^{\text{n}}}$$

Where Q_t_ = the rate of the drug in time t, A = the nanoparticles constant incorporating geometric structure feature, and n = the release exponent that indicates the release rate mechanism.

The zero-order model, frequently referred to as the ideal kinetics model, serves to illustrate the behavior of substances that are released steadily while preserving a uniform concentration level. This model serves to elucidate the mechanisms by which the drug is liberated from coated dosage forms, particularly those exhibiting low solubility in aqueous environments, as well as from osmotic delivery systems^23^. , where a plot of drug release (%) against time is performed using Eq. (2)

2 $${{\text{Q}}_{\text{t}}}={{\text{K}}_0}{\text{t}}$$

Where Q_t_ = the rate of drug released in time t, K_0_ = zero-order model constant unit of inverse time.

### Microbiological analysis

A variety of human infections were utilized to qualitatively assess the antimicrobial efficacy of samples BCuSr 0%, BCuSr 5%, BCuSr 10%, BCuSr 0% + Cipro, BCuSr 5% + Cipro, and BCuSr 10% + Cipro. The studied microbiological cultures included gram-positive bacteria such as *Staphylococcus aureus ATCC 6538 and Bacillus cereus ATCC-6629*, gram-negative bacteria like *Escherichia coli ATCC 25,922 and Klebsiella pneumoniae ATCC-10,031*, as well as the pathogenic yeast *Candida albicans ATCC 10,231.* The microbial species were obtained from the Northern Utilization Research and Development Division of the US Department of Agriculture located in Peoria, Illinois, USA (NRRL), as well as from the American Type Culture Collection (ATCC) based in Rockville, MD, USA. The microbial cultures were prepared using fresh overnight broth cultures and incubated at 37 °C in a nutrient broth medium. Following the preparation and adjustment of the inoculum volume for this pathogenic strain to approximately 0.5 McFarland standard (1.5 × 10^8 CFU/ml), a 25.0 µl inoculum volume from each microorganism strain was individually introduced onto plates containing 20.0 ml of sterile nutrient agar (NA) medium^24^. Additionally, each 100.0 ml conical flask containing 25.0 ml of sterile nutrient broth (NB) medium was inoculated with 25.0 µl of the bacterial and fungal suspensions individually. Upon cooling and solidification of the medium within a 0.9 cm well of the infected agar plates—previously prepared utilizing the Well Diffusion Method and a 1.0 cm cork borer—each well was systematically filled with 100.0 µl of each sample^25^. The antibacterial efficacy was quantified by assessing the diameter of the inhibition zone in millimeters after a 24-hour incubation at 37 °C, followed by a one-hour refrigeration phase to facilitate additional sample diffusion.

### The MTT based cytotoxicity assay

The cytotoxicity evaluation was performed in accordance with the ISO 10993-5 standards, which guide the biological assessment of medical devices, particularly in vitro cytotoxicity testing^26^.

The tetrazolium dye (MTT) assay was used to assess the cytotoxicity^27^. The ATCC (American Type Culture Collection) in the United States provided the human bone osteosarcoma cells (MG-63) and bone marrow stromal cells, which were cultivated in triplicate on 24-well culture plates for 48 h at a density of 1 × 105 cells/ml. The absorbance of related cells and control cells was divided to determine the percentage of cell viability. Additionally, using the dose-response curve calculation, the half maximal inhibitory concentration (IC50) was determined for Silicon (2022) 14:11171–11,180. More details can be found in reference^28^. The National Research Centre in Dokki, Egypt’s medical research ethics committee accepted and reviewed the study protocol in accordance with its ethical standards. Number of approval: 11,410,823.

### Statistics analysis

For *n* = 3, all data were presented as means ± standard deviation (SD), and the standard analysis of students’t-test was used for analysis. A significance level of *p* < 0.05 is established.

## Results & discussion

### TEM characteristic

**Fig. 1:**
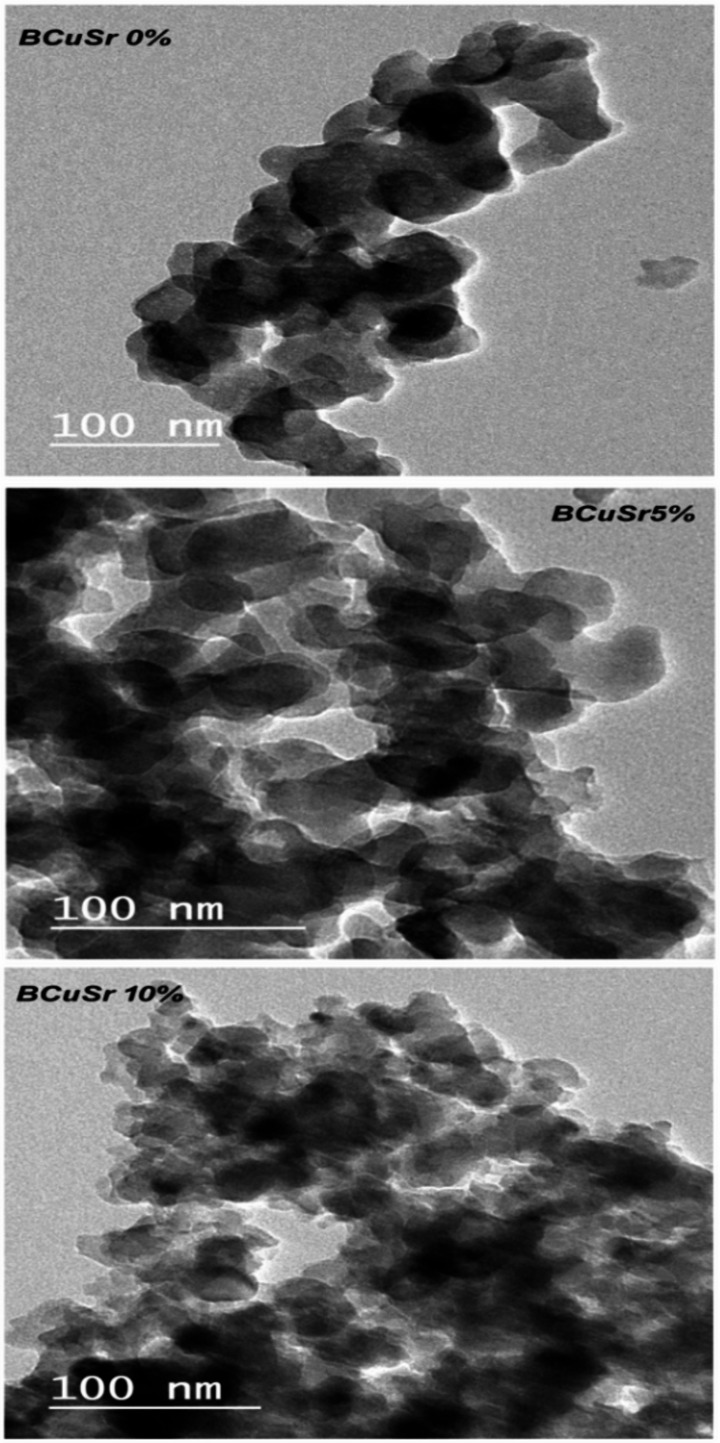
The TEM photos of prepared sol-gel for the bioactive glasses samples BCuSr 0%, BCuSr 5% and BCuSr 10%.

The image illustrates the transmission electron microscopy (TEM) analysis of all prepared glasses, as presented in Fig. 1. The dimensions of all glass particles are under 100 nm; specifically, the diameter of Nano-bioactive glass ranges from 30 to 50 nm, while the structure of bioactive glass comprises nanoparticles of varying sizes and irregular geometries. Meanwhile, Ali Can Özarslan^29^ reported that the co-incorporated bioactive biosilica glasses were prepared via the modified melt-quenching approach, which does not obtain nano-size reverse sol-gel method.

### XRD diffraction

**Fig. 2:**
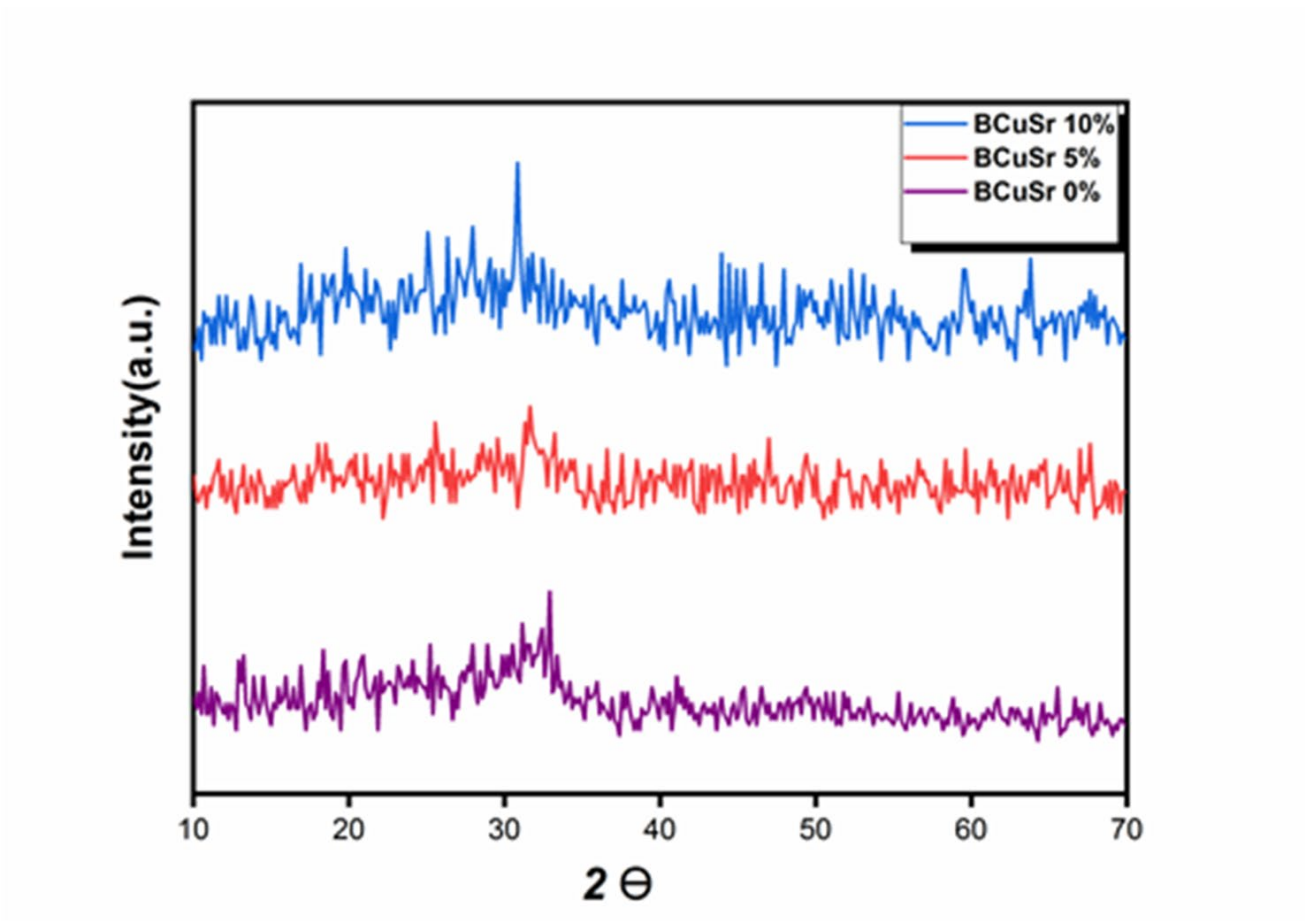
The XRD Pattern for the samples BCuSr 0%, BCuSr 5% and BCuSr 10%.

Due to the crystallization of bioactive glass occurring around 550 °C, the heat treatment of samples results in the formation of amorphous bioactive glass (BG), as illustrated in Fig. 2.The diffractogram exhibited a subtle peak at 2θ = 32°, attributed to calcium silicate (Ca2SiO4) (JCPDS # 29–0369)^16^. The amorphous nature of the synthesized BG has been validated. Ali Can Ozarslan demonstrated that the silicate glasses produced from all prepared samples exhibited amorphous structures^8^. The subdued peak can be ascribed to incomplete crystallization that took place during the calcination process at 550 °C. The XRD spectra for all Cu-doping BG samples reveal the absence of any distinct diffraction peaks, presenting only a broad hump around 2θ = 32°, which signifies their amorphous characteristics. Due to the absence of CuO or other copper phases in the XRD patterns, it is inferred that solely Cu^2+^ ions are present within the silicate glass network^28,30^. As varying amounts of Strontium are incorporated into the bioactive glass, no new diffraction peaks are observed in the XRD patterns of BCuSr 5% and BCuSr 10% relative to BCuSr 0%. By substituting some Calcium with Strontium in the crystal lattice of BCuSr 0%, the diffraction peaks of BCuSr 5% and BCuSr 10% shift to lower angles. Furthermore, as the concentration of Sr rises, the extent of the shift amplifies. At approximately 32°, the pronounced twin peaks ultimately merge and unify into a singular peak. The ionic radius of Sr2+ (1.12) is significantly larger compared to that of Ca^2+^ (0.99).

### FTIR characteristic

**Fig. 3:**
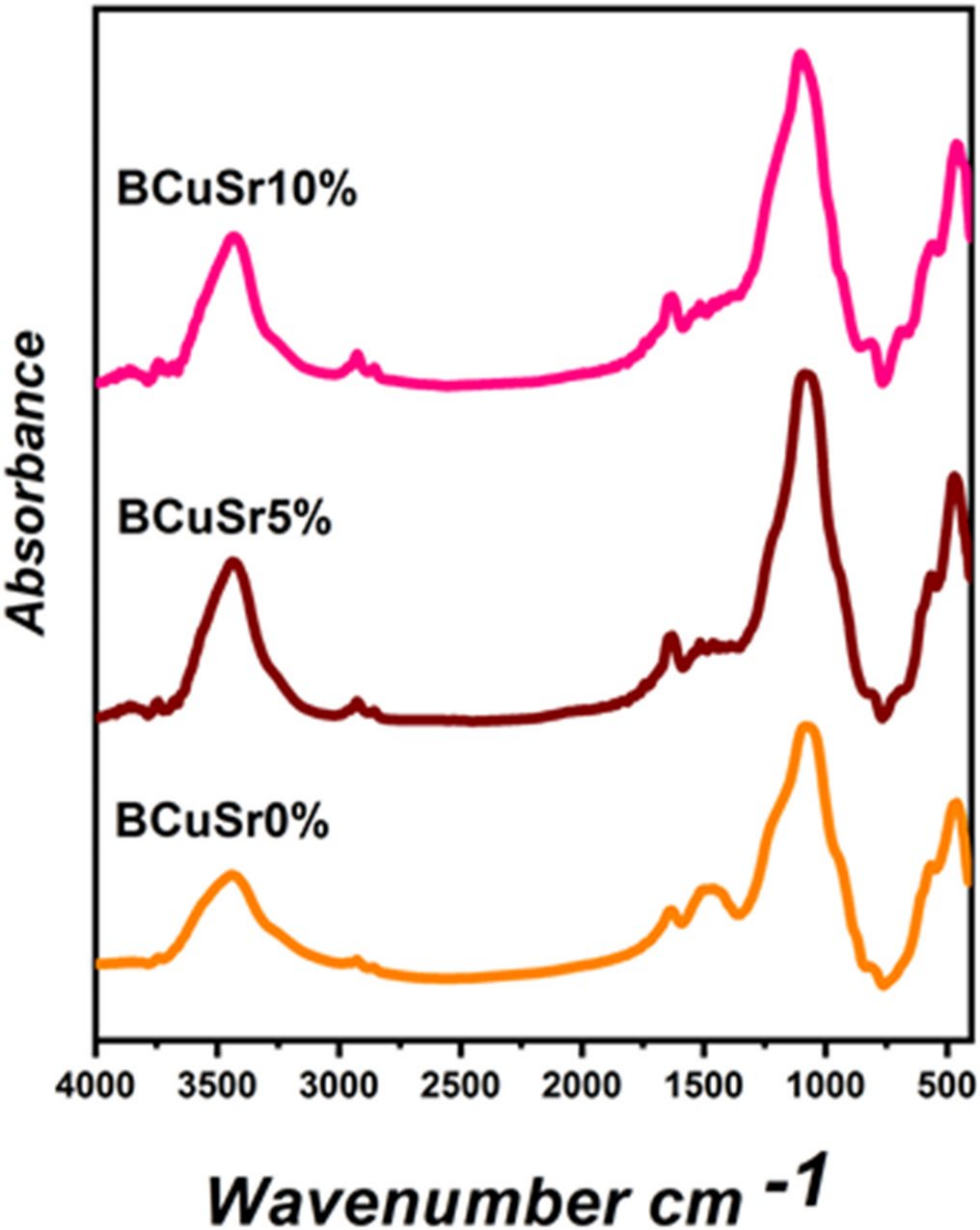
FTIR Characteristics for the samples BCuSr 0%, BCuSr 5% and BCuSr 10%.

The FTIR spectra of the glass samples are presented in Fig. 3. In all glass samples, the bands observed in the 1000–1200 cm^− 1^ range correspond to the asymmetric stretching vibration of Si-O-Si, whereas the bands found in the 725–800 cm^− 1^ range signify the symmetric stretching vibration of Si-O-Si^31,32^. The peak observed at 1250 cm^− 1^ corresponds to the asymmetric and symmetric stretching modes of Si-O-Si. The stretching mode of the OH group in H_2_O, characterized by a wide spectrum of hydrogen bond strengths, appeared at 3500 cm^− 1^^33^. The bending of Si-O-Si contributes to a medium band observed at 450 cm^− 1^. P-O bending vibrations are characterized by two main vibrational bands located at 590 and 610 cm^− 1^. By incorporating CuO and SrO into the blank sample (BCuSr 5% and BCuSr 10%), it is observed that the spectral properties remain consistent in both location and intensity across all characteristic IR bands. The samples contain a significant quantity of silica as the primary component^28^. The incorporation of SrO preserves a significant degree of bioactivity, reinforcing the idea that SrO enhances the active properties of the prepared BG^30^. Nonetheless, the influence of SrO remains undetectable in the FTIR analysis. Furthermore, this aligns with the findings of Ali Can Özarslan, who observed that the co-incorporation of Sr–Cu did not notably alter the FT-IR spectra of biosilica glasses^8^.

### Textural analysis of glass nanoparticles

**Fig. 4:**
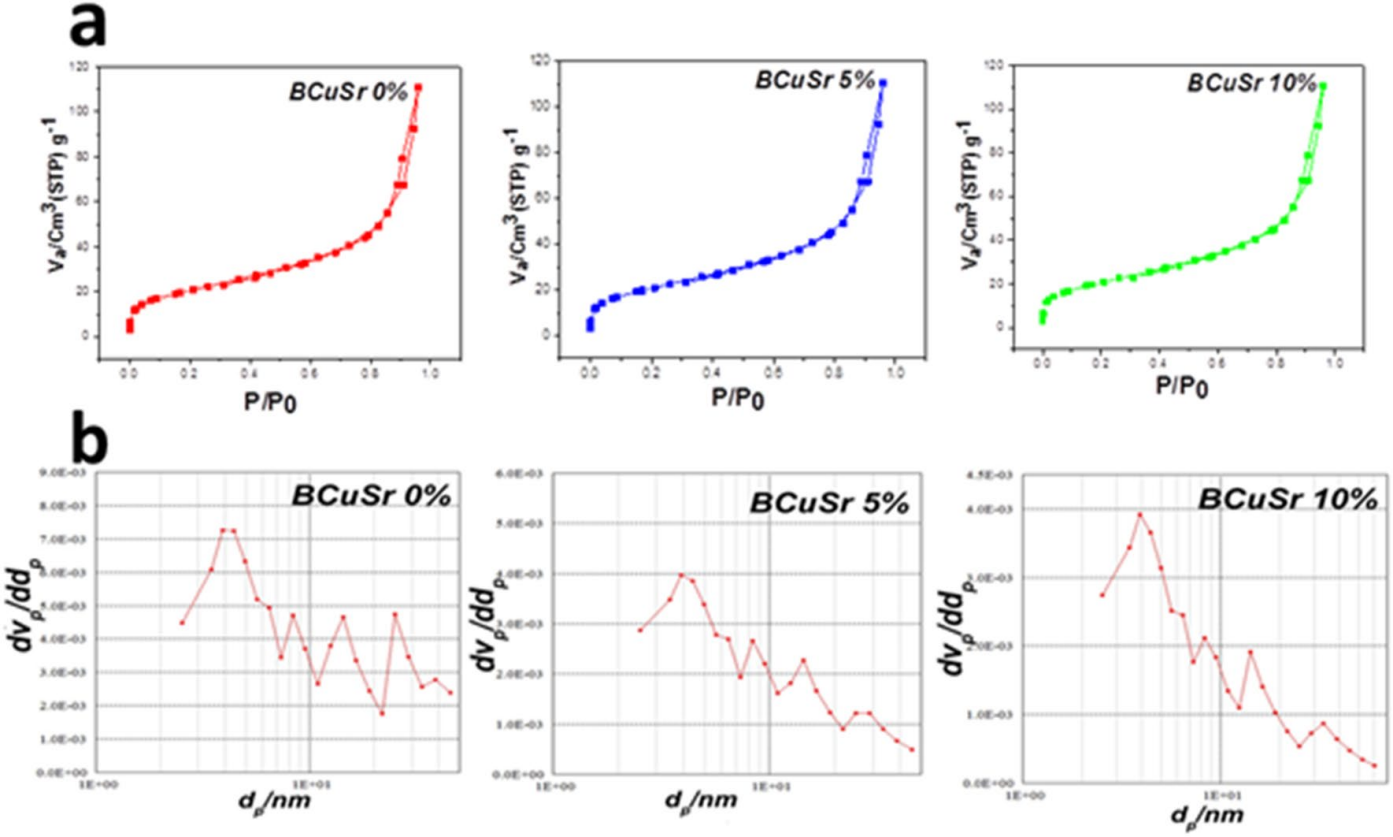
(**a**) Isothermal curves and (**b**) pore size distribution for the samples BCuSr 0%, BCuSr 5% and BCuSr 10%.

Assessing specific surface area is crucial for drug delivery systems as it determines factors like pore size and volume, which influence the properties and dynamics of drug loading and release from porous nanomaterials^12,34^. The nitrogen adsorption–desorption isotherms and pore size distribution of the BCuSr 0%, BCuSr 5% and BCuSr 10% samples are shown in Fig. 4. Based on the IUPAC classification, all samples exhibited mesoporous structures characterized by the type IV isotherm, as illustrated in Fig. 4a. Furthermore, as illustrated in Fig. 4b, the pore width varies from 1 to 10 nm, and the H3-type hysteresis loop is indicative of the pore structure for nanomaterial’s (featuring irregular, slit-shaped, and interconnected pores). Table 2 presents the specific surface area, pore diameter, and total pore volume of the produced samples. Table 2 indicates that the doped of CuO nanoparticles into BG resulted in a decrease in specific surface area values, falling to 31 m^2^/g for BCuSr 10%, in contrast to the 74 m^2^/g observed for BCuSr 0% alone^22^. In an effort to elucidate whether the presence of copper could influence them in any manner. In summary, most results indicate a reduction in the specific surface area and structural configuration of mesoporous materials in Cu-doped MBGs as the copper content rises. The incorporation of Cu^2+^ into the glass network is observed to decrease specific surface area, pore volume, and mesoporous size when compared to the undoped system, as demonstrated in a study by Wu et al.l^35^. Upon incorporating the strontium element into the bioactive glass, we observed no discernible effects.

The average pore diameter of the nanoparticles for BCuSr 0%, BCuSr 5%, and BCuSr 10% was measured at 9.17, 6.95, and 7.87 nm, respectively. The incorporation of doped CuO and SrO into Nano-bioactive glass led to a slight reduction in pore size. For BCuSr 0%, BCuSr 5%, and BCuSr 10% nanoparticles, the total pore volume measures 0.17, 0.07, and 0.06 (cm^3^/g), respectively. Research indicates that there is typically a reduction in overall pore volume as the surface area expands^24^.

**Table 2:** Pore diameter, specific surface area, and total pore volume for the samples BCuSr 0%, BCuSr 5% and BCuSr 10%.

Samples	Pore diameter (nm)	Specific surface area (m^2^/g)	Total pore volume (cm^3^/g)
BCuSr 0%	9.17	74	0.17
BCuSr 5%	6.95	40	0.07
BCuSr 10%	7.87	31	0.06

### In vitro bioactivity evaluation

#### Scanning electron microscope coupled with energy dispersive X-Ray (SEM/EDX)

**Fig. 5:**
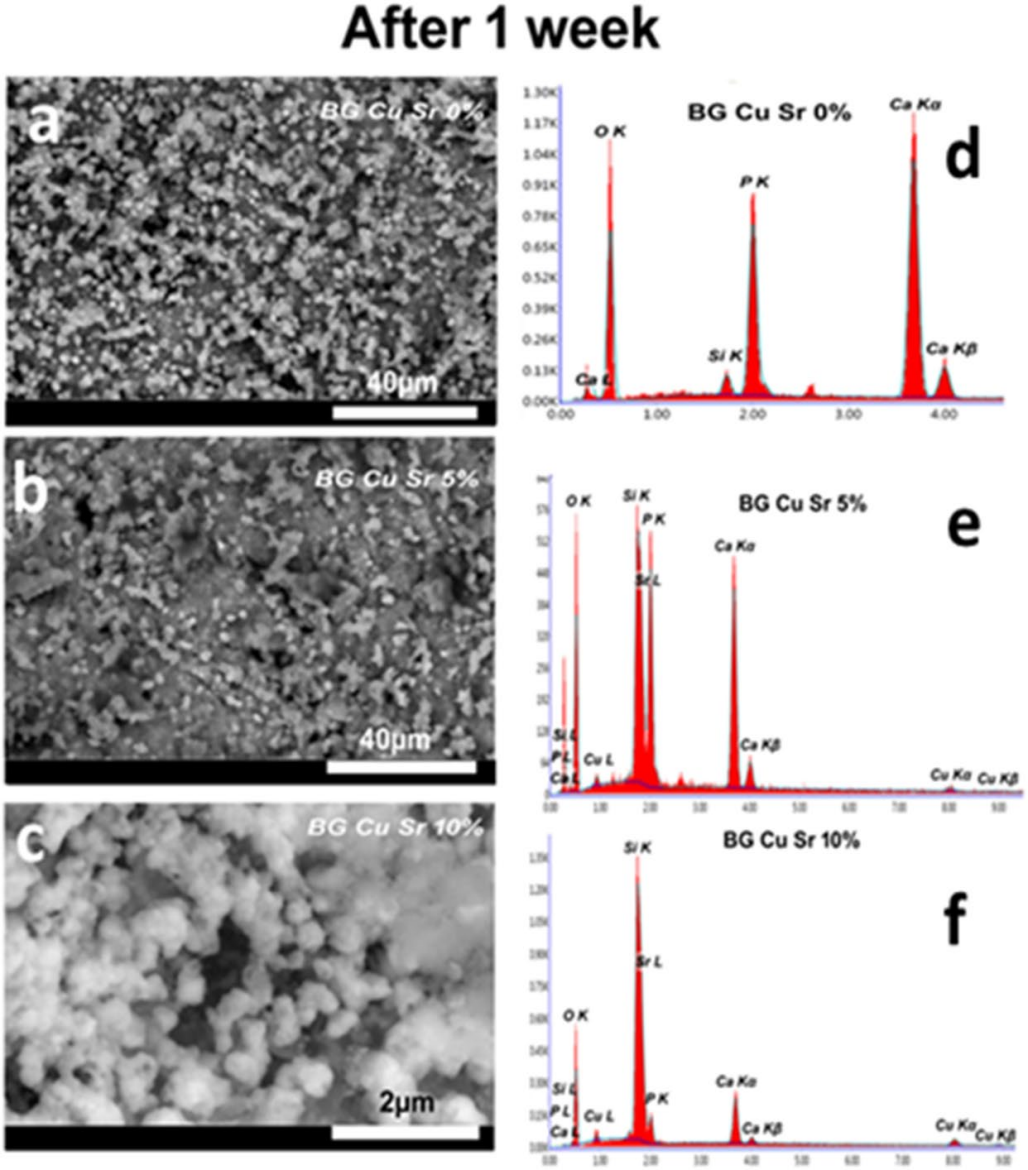
The SEM micrographs for the samples (**a**) BCuSr 0%, (**b**) BCuSr 5% and (**c**) BCuSr 10% glasses after immersion (7 days) in the SBF and the EDX analysis of their surfaces (**d**) BCuSr 0%, (**e**) BCuSr 5% and (**f**) BCuSr 10%.

**Fig. 6:**
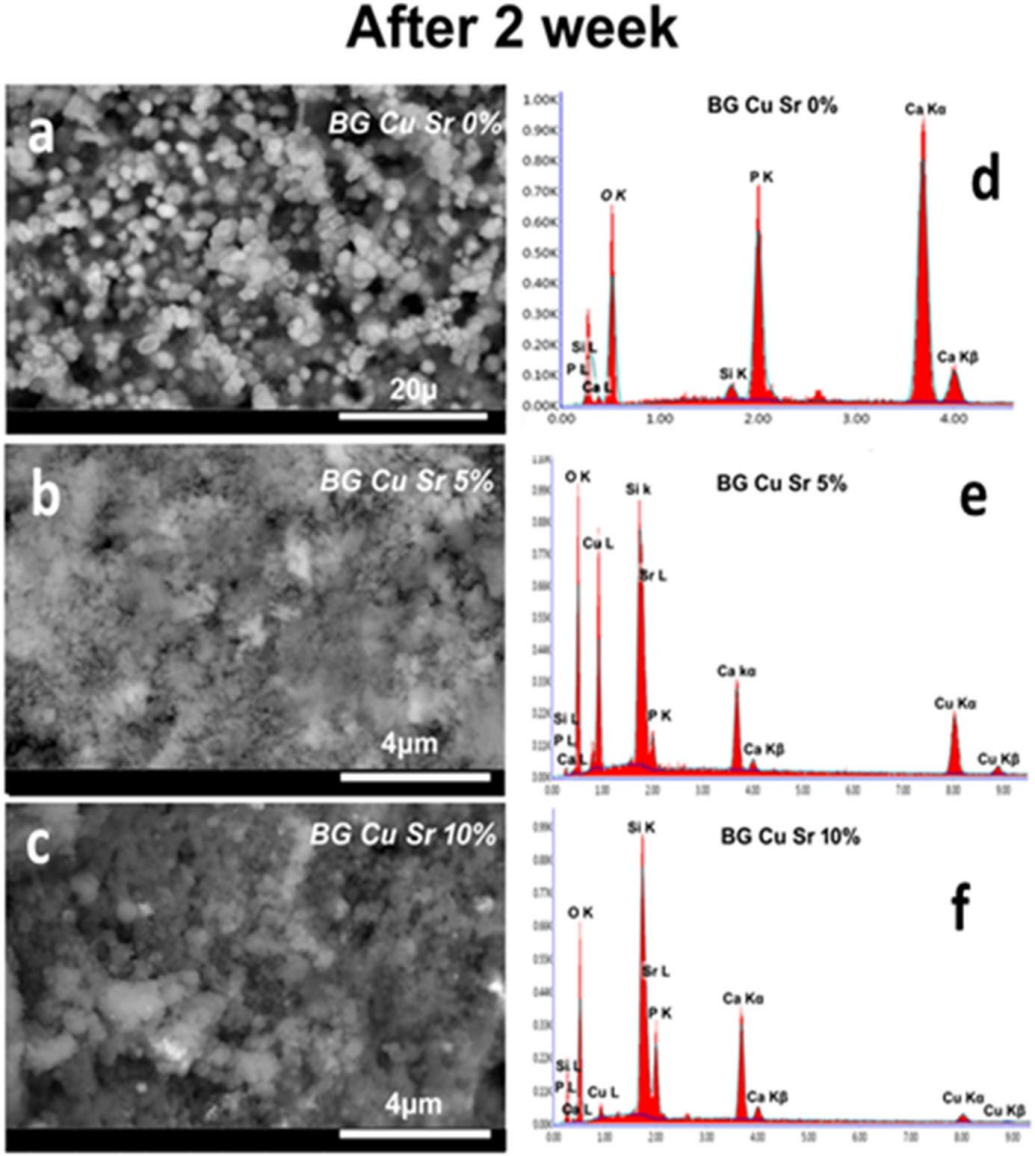
The SEM micrographs for glasses for the samples (**a**) BCuSr 0%, (**b**) BCuSr 5% and (**c**) BCuSr 10% after immersion (14 days) in the SBF, and EDX analysis of their surfaces (**d**) BCuSr 0%, (**e**) BCuSr 5% and (**f**) BCuSr 10%.

Figures 5 and 6 present SEM micrographs of all glass disk samples following immersion in SBF (simulated body fluid) for 7 days and 14 days. The figures reveal the precipitation of spherical particles across the entire surface area of the glasses, indicating the formation of a hydroxyapatite layer (HAp). Prolonged soaking of the samples leads to an increased thickness of the hydroxyapatite layer. The dissolution of Ca and P ions in the SBF solution, leading to their precipitation, is responsible for the formation of the HAp layer. The significant establishment of the Ca–P layer on the glass surface could be linked to the remarkable progression of the HAp layer; the particle sizes of the samples vary from 30 to 50 nm. CuO does not significantly influence the propagation of the HAp layer^36^ while the inclusion of Cu alters the shape and size of the apatite crystals, yet it does not inhibit the formation of the hydroxyapatite layer^37^. The increase of SrO amount leads to enhance in the bioactivity of the glasses^30^. In vitro studies indicate that Sr-doped glasses exhibit enhanced bioactivity compared to undoped systems, leading to a more rapid deposition of the apatite layer on their surface. This phenomenon is probably due to the release of Sr ions into the dissolution medium at crucial concentrations ranging from 1 to 5 ppm. Moreover, in line with Cacciotti’s assertion, the marginally greater radius of Sr compared to Ca boosts the reactivity of Sr-doped BGs both in vitro and in vivo. This enhancement occurs through the expansion of the silica network, which subsequently elevates the ion dissolution rate^37^. In contrast to Ali Can Ozarslan et al. found that the morphology of the glasses under investigation did not significantly change when Sr and Cu were added to the glass structure^9^. On the other hand, above a specific concentration of strontium ion, the substitution of Sr for Ca in high-phosphate bioactive glasses directly inhibits the formation of the apatite layer, which typically involves the transformation into hydroxyl carbonate apatite (HCA) after the deposition of an octacalcium phosphate (Ca_8_H_2_ (PO_4_)_6_-5H_2_O) phase. Actually, an excessively high Sr concentration delays the deposition of HCA by inhibiting the octacalcium phosphate phase^37^. The analysis of EDX presented in Table 3 corroborates these results. The Ca/P ratios of the HAp layer closely resemble the Ca/P ratio of mineral hydroxyapatite, which is 1.67. EDX analysis additionally reveals the presence of other elements. The Ca/P ratio of the HAp layer developed on the glass surface over a period of 7 days is measured at 1.82, 1.65, and 1.61 for BCuSr concentrations of 0%, 5%, and 10%, respectively. Conversely, the Ca/P ratios after 14 days were 1.9, 2.3, and 1.3 for BCuSr 0%, BCuSr 5%, and BCuSr 10%, respectively. The ratios are comparable to those found in natural hydroxyapatite. Ultimately, the complete coverage of the glass surface with hydroxyapatite layers demonstrates the potential of these materials for application in bone treatment and defect regeneration^38^.

**Table 3:** The EDX analysis for all for the glasses samples BCuSr 0%, BCuSr 5% and BCuSr 10%.

After 7 days atomic %						After14 days atomic %				
Samples	Ca	*P*	Cu	Sr	Ca/*P*	Ca	*P*	Cu	Sr	Ca/*P*
BCuSr0%	14.93	8.17	0	0	1.82	17.35	9.13	0	0	1.9
BCuSr5%	10.9	10.78	0.87	4.03	1.65	5.52	2.4	0.69	8.84	2.3
BCuSr10%	5.85	3.62	2	8.80	1.61	8.38	6.46	1.92	4.16	1.3

#### Ion release analysis

Throughout different time intervals, the levels of calcium, phosphorus, silica, copper, and strontium ions released from samples in SBF solution are assessed. Changes in SBF ion concentrations indicate the formation of hydroxyapatite on sample surfaces.

**Fig. 7:**
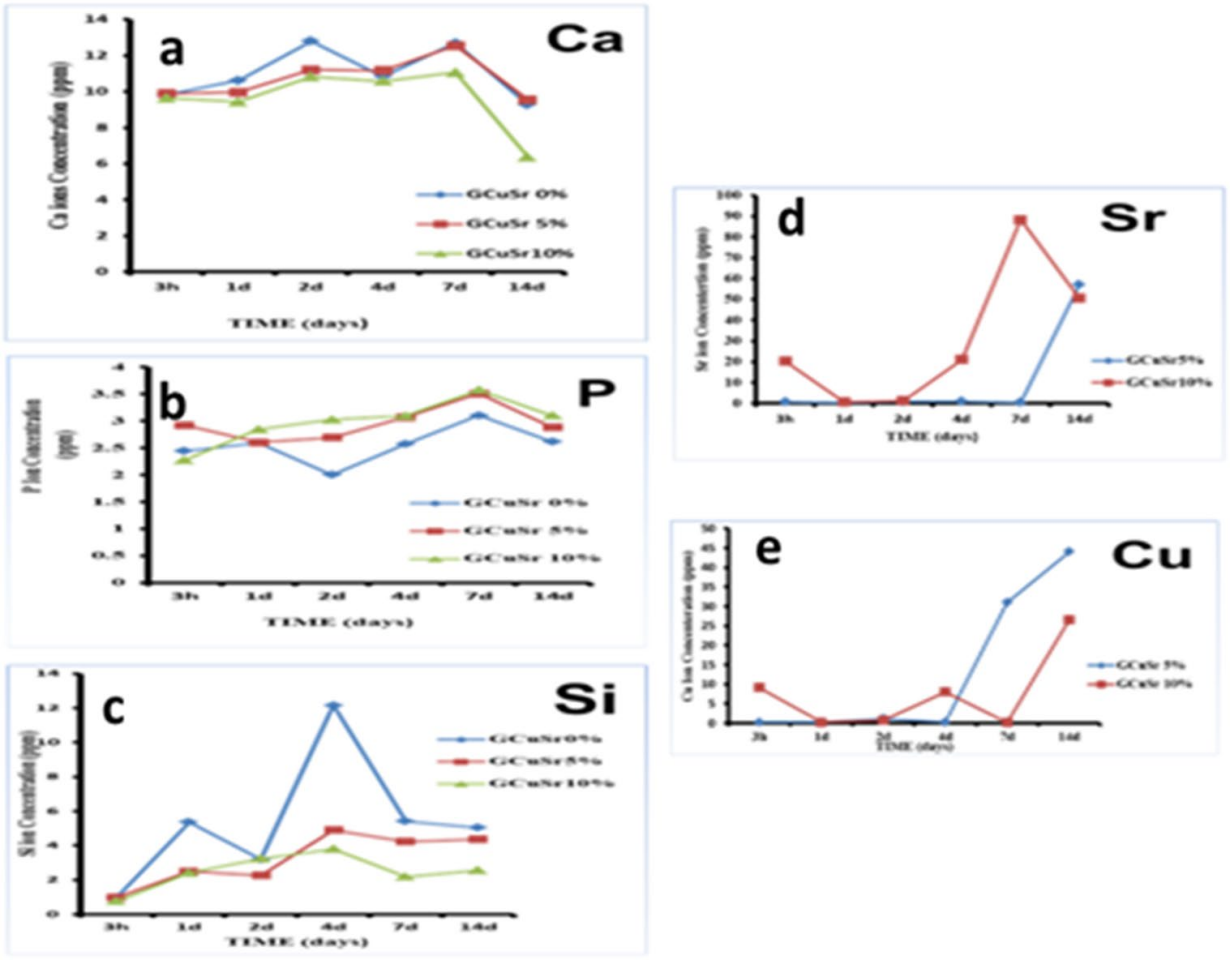
The Ions concentrations in SBF solution at different periods of soaking time for (**a**) Ca, (**b**) P, (**c**) Si, (**d**) Sr and (**e**) Cu for BCuSr 0%, BCuSr 5% and BCuSr 10%.

The dissolution profiles of the prepared BCuSr 0%, BCuSr 5%, and BCuSr 10% samples in SBF at different time intervals are illustrated in Fig. 7. The levels of the elements Ca, P, Si, Cu, and Sr are interrelated with it.

As illustrated in Fig. 7a, the concentration of calcium ions rises steadily during the initial two days of immersion in SBF solution, reaching a maximum of 12.8 ppm for BCuSr 0%, while BCuSr 5% and BCuSr 10% show concentrations of 12.5 and 11.2 ppm, respectively, over a period of seven days. After a 14-day immersion period, the calcium ion levels for BCuSr 0%, BCuSr 5%, and BCuSr 10% decreased to 9.2, 9.5, and 6.3 ppm, respectively. The interaction of calcium ions with H^+^ or H_3_O^+^ ions in the solution may explain the increase in ion concentration observed during the initial two and seven days. The observed decrease in calcium ion concentration may be attributed to the deposition of Ca^+ 2^ ions on the surface of the materials, driven by the attraction of negative charges from Si–OH groups^24^.

Figure 7b illustrates the variation of phosphorus ions in SBF as a function of different soaking durations for the samples. The phosphorus ion concentration in all prepared samples shows a notable decline after two days of soaking, achieving minimum values of 2, 2.6, and 3 ppm for BCuSr 0%, BCuSr 5%, and BCuSr 10%, respectively. After 14 days of immersion, the phosphorus ion concentrations in each sample converged, measuring 2.6, 2.8, and 3 ppm for BCuSr 0%, BCuSr 5%, and BCuSr 10%, respectively. The presence of calcium and phosphate ions in the simulated body fluid solution is linked to two contrasting processes: their concentration in the solution rises as they are released from the glass network, while it diminishes as they are utilized in the formation of the apatite layer. The observed change in Ca concentration indicates that, in the initial phases of soaking, there is a more significant release of Ca ions compared to their uptake. The decrease in phosphorus ions, especially during the early soaking periods, indicates that a greater amount of phosphorus is utilized in the hydroxyapatite synthesis process compared to what is liberated from the sample^24^.

Silicon ions are absent in the priming fluid of SBF. When Si–O–Si bonds are disrupted due to interactions between the glass lattice and SBF liquid, soluble Si (OH)_4_ is released, leading to the presence of silicon ions in SBF.

Figure 7c illustrates that the silicon concentration in the solution for the BCuSr 0%, BCuSr 5%, and BCuSr 10% samples increases rapidly, achieving peak values of 12.3, 4.9, and 3.8 ppm, respectively, after a duration of 4 days. The silicon ion concentration for all samples is remarkably similar at the conclusion of the soaking period, measuring 4.6, 4.2, and 3 ppm for BCuSr 0%, BCuSr 5%, and BCuSr 10%, respectively. The dissolution of the outer silica layers of the network marks the initial phase of the release of silicon ions.

Upon the cleavage of Si–O–Si bonds, silanols (Si–OH) emerge at the interface between glass and solution, leading to the dissolution of solid silica into the solution as monosilicic acid Si(OH)_4_^24^.

As illustrated in Fig. 7d, the levels of Sr released into the solution for the BCuSr 5% and BCuSr 10% samples are presented. The BCuSr 10% exhibited a higher initial release compared to BCuSr 5%, attributed to its elevated strontium ion content. After 14 days, the maximum values reached were 57 ppm for BCuSr 10% and 50 ppm for BCuSr 5% samples, respectively. The findings of the current study indicate that the release of Sr^2+^ ions increased with a higher amount of strontium replacing calcium, with the most significant release occurring during the first week of immersion.By substituting the smaller ionic radius of Ca2 + ions (0.99 Å) with the larger ionic radius of Sr^2+^ ions (1.13 Å), this issue may be addressed^39^. The implications of replacing calcium with strontium in dental materials continue to be a topic of discussion and investigation. Further investigation is essential to elucidate the role of strontium in the prevention of carious lesions. Many studies have shown that the release of Sr2 + ions enhances tooth remineralisation, reduces osteoclastic bone resorption, and boosts osteoblastic bone formation^40^.

In Fig. 6e, the concentration of Cu released in the solution for the BCuSr 5% and BCuSr 10% samples shows that the BCuSr 10% exhibited a higher initial release compared to BCuSr 5%, due to its greater copper content. This concentration peaked at 44 ppm for BCuSr 10% and 26 ppm for BCuSr 5% after 14 days of soaking. The slower release of Cu is likely attributed to co-precipitation in HCA or a modification in the dissolving mechanism. At the highest glass concentrations and extended dissolution periods, the release of copper ions appears to reach a saturation point; however, it increases alongside both glass concentration and dissolution duration^40^. The release of therapeutic dosages of Cu^2+^ ions from bioactive glasses has been clearly shown to enhance the healing process of wounds by promoting angiogenesis, leading to the synthesis of vascular endothelial growth factor (VEGF), and providing an antimicrobial effect^40^.

Overall, the Sr ions release rate is marginally higher than the Cu release rate for each system, most likely as a result of the Sr ions’ larger ionic radii (1.16 Å) compared to the Cu ions’ (0.73 Å)^30^. For every glass under investigation, Ali Can Ozarslan et al.‘s findings also showed a clear link between a low _Ea−Si_ value and a quick ionic release. Additionally, the trends for all the glass under investigation were similar; as the percentage Cu content increased, the ion release for Si, Ca, Na, P, and Sr decreased. As anticipated, the ion release for Cu is completely the opposite^9^.

### Antibacterial effect

**Fig. 8:**
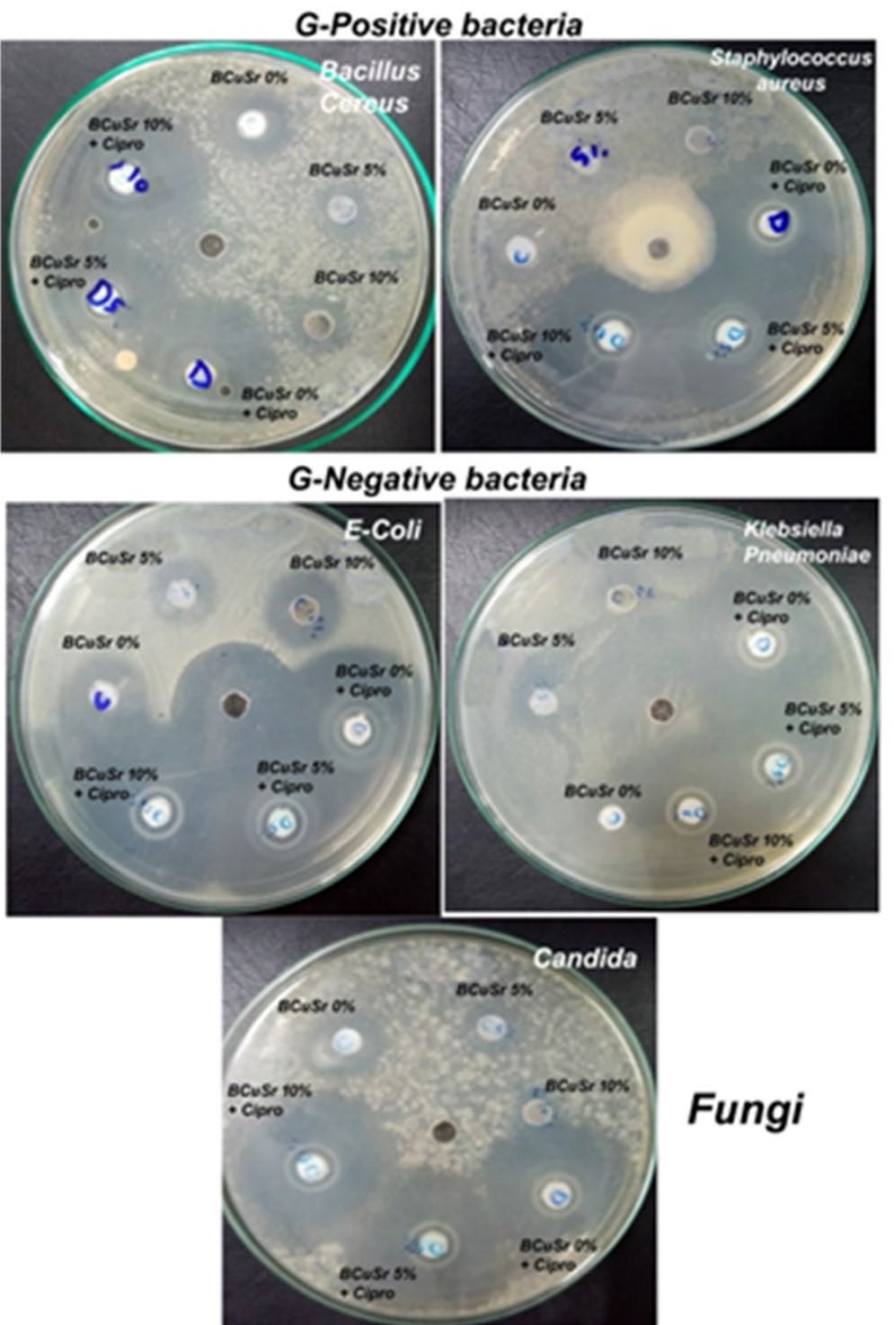
The Antimicrobial effects of the samples BCuSr0%, BCuSr5% and BCuSr10% against different microorganism with and without antibiotics.

One of the most crucial characteristics of bioactive glasses is their antibacterial activity, which helps to heal and regenerate the damaged area while establishing a bacterial-free environment. The antibacterial activity of the produced samples BCuSr0%, BCuSr5% and BCuSr10% is tested both with and without antibiotics. The inhibition zone diameter (mm) of samples against different microbes is shown in Table 4; Fig. 8. Gram-positive bacterial strains (*Bacillus cereus ATCC-6629 and Staphylococcus aureus ATCC-6538*), and gram-negative bacterial strains (*Escherichia coli ATCC-25922 and Klebsiella Pneumoniae ATCC-10031*) as well as antifungal activities (*Candida albicans ATCC-10231*) are tested. With or without ciprofloxacin, every sample shows the presence of an inhibitory zone against every bacterium that was analysed. Because CuO and SrO have antibacterial properties, we noticed that there is an inhibitory zone with varying sizes surrounding samples.

Numerous investigations examining the antibacterial properties of copper ions utilize both Gram-positive and Gram-negative bacteria to elucidate how their distinct cell wall architectures influence metal susceptibility and antibacterial effectiveness. Copper serves as an exceptional antimicrobial agent and is integral to the processes of bone formation and healing. Gram-positive bacteria that exhibit a strong attraction to copper ions and copper-containing compounds, such as *Bacillus subtilis (B. subtilis)* and *Staphylococcus aureus*, possess a significant number of amines and carboxyl groups on their cell surfaces. Moreover, the redox properties of copper within the periplasmic space play a significant role in its antibacterial efficacy against Gram-negative bacteria. Cu-NPs induce denaturation of intracellular proteins, leading to interactions with sulphur-containing chemicals and biomolecules. Nonetheless, it is widely recognized that copper ions penetrate the cell membrane following their gradual release from metallic surfaces and copper nanoparticles. This facilitates direct engagement with the functional groups of intracellular proteins and nucleic acids. Consequently, it is believed that both the nanoparticles and the released copper ions contribute to the antibacterial properties of Cu-NP. The positively charged copper ions are attracted to the negatively charged cell membranes, similar to the behaviour of Cu-NPs^41^. According to Ali Can Ozarslan et al., Sr-incorporated 50S6P degradable glass and Sr and Cu co-incorporated 50S6P degradable glasses showed good bioactivity properties at a reasonable level, making them good candidates for bone tissue engineering applications. Because of their promising features (possible cellular and antibacterial activities), they may be recommended as bone graft materials in preclinical and subsequent clinical studies^8^.

After the incorporation of the drugs into the prepared samples, there was a notable enhancement in the diameter of the inhibition zones against a range of microbial strains.This suggests that following its application to the prepared samples, the drug preserved its antibacterial efficacy. The span of the inhibitory zones measures between 14 and 50 mm. The sample exhibiting the most significant inhibitory zones across all tested microorganisms is the one that incorporated a combination of CuO and SrO with the drugs. Loading ciprofloxacin into nanoparticles (BCuSr0% + Cipro, BCuSr5% + Cipro, and BCuSr10% + Cipro) enhances the inhibitory activity of the samples while maintaining the drug’s antibacterial efficacy. By disrupting the structural integrity of the cell wall, ciprofloxacin induces lysis in microorganisms. Transitioning from the outer membrane to the inner membrane, this disruption occurs sequentially. Upon exposure to ciprofloxacin, there is a notable loss of protein and lipopolysaccharide in the outer membrane, accompanied by alterations in the banding patterns of the outer membrane proteins^24^. Furthermore, ciprofloxacin reduces the levels of calcium and magnesium within cell envelopes. Ciprofloxacin is thought to disrupt and eliminate organic components by displacing essential metal cations in the outer membrane^42^. Generally, Ciprofloxacin functions by inhibiting protein synthesis and facilitating the intracellular elimination of microorganisms.

**Table 4:** The Inhibition zone diameter (mm) for the samples BCuSr 0%, BCuSr 5% and BCuSr 10%.

Pathogens	Gram Positive		Gram Negative		Fungi
	Bacillus cereus ATCC-6629	Staphylococcusaureus ATCC-6538	Klebsiella pneumoniae ATCC-10,031	Esherichia coli ATCC- 25,922	*Candida albicans* ATCC-10231
BCuSr0%	39	45	44	50	18
BCuSr5%	38	42	39	46	15
BCuSr10%	37	46	33	48	17
BCuSr0%+cipro	14	14	19	26	19
BCuSr5%+cipro	15	16	16	24	18
BCuSr10%+cipro	16	18	17	25	15

### Delivery system for Ciprofloxacin

#### Drug loading efficiency

**Fig. 9:**
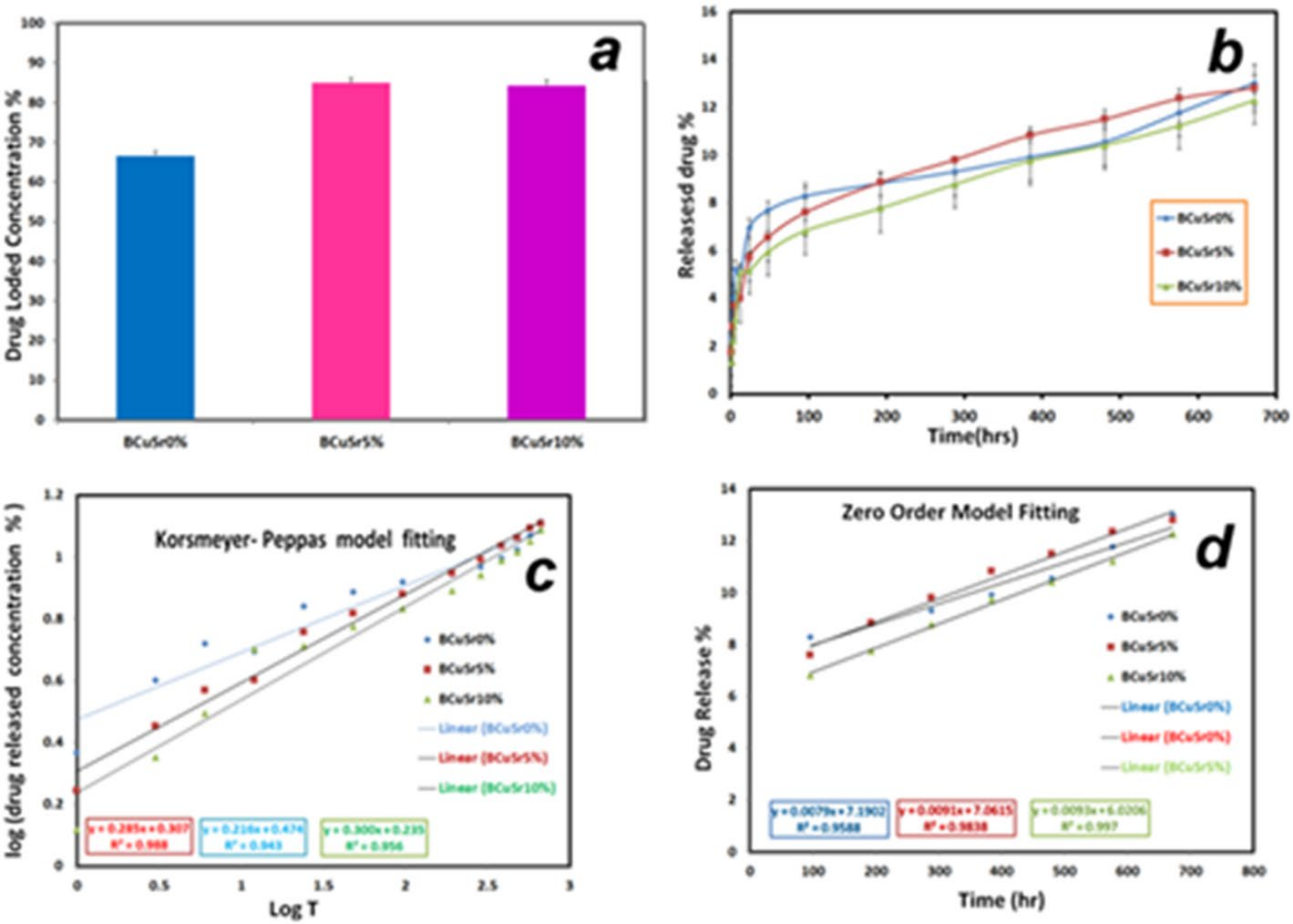
(**a**) The loaded drug, (**b**) The drug cumulative concentration release, (**c**) The fitting of drug data with Korsmeyer-Peppas model and (**d**) The fitting of drug data with zero order models.

This investigation utilizes ciprofloxacin due to its broad-spectrum antibacterial properties. This is a second-generation fluoroquinolone antibiotic, categorized as a Class II/IV medication according to the Biopharmaceutical Classification System (BCS), utilized for the treatment of bacterial infections^19^. The loading capacity of ciprofloxacin on BCuSr0%, BCuSr5%, and BCuSr10% is illustrated in Fig. 9a. The findings indicate that ciprofloxacin has been effectively incorporated into all samples. A significantly higher percentage of ciprofloxacin (85 ± 1.66%) is adsorbed by BCuSr5% in comparison to BCuSr0% and BCuSr10% (66.5 ± 1.19% and 84.35 ± 0.28%, respectively), as illustrated in Fig. 9a.The tendency of silicon groups in the glass network to hydrolyze into SiOH groups, which can subsequently form hydrogen bonds with the functional groups of ciprofloxacin molecules, accounts for the significant drug adsorption^19^.

#### Drug release profile

Figure 9b depicts the cumulative release profile of ciprofloxacin from the glasses as a function of time. The drug release profile indicates a significant rapid release phase within the initial 24 h, followed by a gradual release phase throughout the rest of the incubation period. For BCuSr0%, BCuSr5%, and BCuSr10% glass, approximately 6.932 ± 0.39%, 5.713 ± 0.23%, and 5.16 ± 1.36% are released in the initial rapid phase, while 13.02 ± 0.78%, 12.81 ± 0.53%, and 12.29 ± 3.28% are released by the conclusion of the subsequent gradual phase, respectively. Moreover, it is important to note that the percentage of drug released from each sample does not show statistical significance. However, the sample BCuSr0% exhibits a greater release compared to the other samples. This phenomenon can be attributed to the hypothesis of electrostatic interaction, which provides an explanation for the observed result, as silanol groups are forming and the surface of the nano-bioglass carries a negative charge in solution. A variety of functional groups have been recognized in ciprofloxacin, and the increase in non-bridging oxygen bonds (NBOs) enhances the Si-OH groups and the negative surface charge of the nano-bioglass. This occurs through the formation of hydrogen bonds with silanol groups, which aids in their adhesion to silica nanoparticles. Certain groups could play a role in imparting a negative charge to the ciprofloxacin molecule. The nano-bioglass surface and ciprofloxacin exhibit electrostatic repulsion due to their negatively comparable charges^18^.

#### Drug released kinetics

To ascertain the mechanism of drug release from the glass, a study on drug release kinetics is carried out. The Korsmeyer-Peppas and the zero order models are two computed models used to determine the in vitro release mechanism of ciprofloxacin. Kinetic equations and the regression coefficient, R^2^, served as the foundation for the data fitting. R^2^ values greater than 0.99(R^2^ > 0.99) indicate that the Korsmeyer-Peppas model as in Fig. 9c fit the data well in both the fast and slow release stages. It describes the release of a drug from a polymeric structure while taking non-fickian mechanisms into account. and the zero order model also fit the date well (R^2^ > 0.99) as in Fig. 9d the process by which a drug is released from a drug delivery device at a steady rate (that is, the same amount of drug is released per unit of time regardless of the amount of drug left in the delivery system)^18,19^.

### In vitro MTT assay

**Fig. 10:**
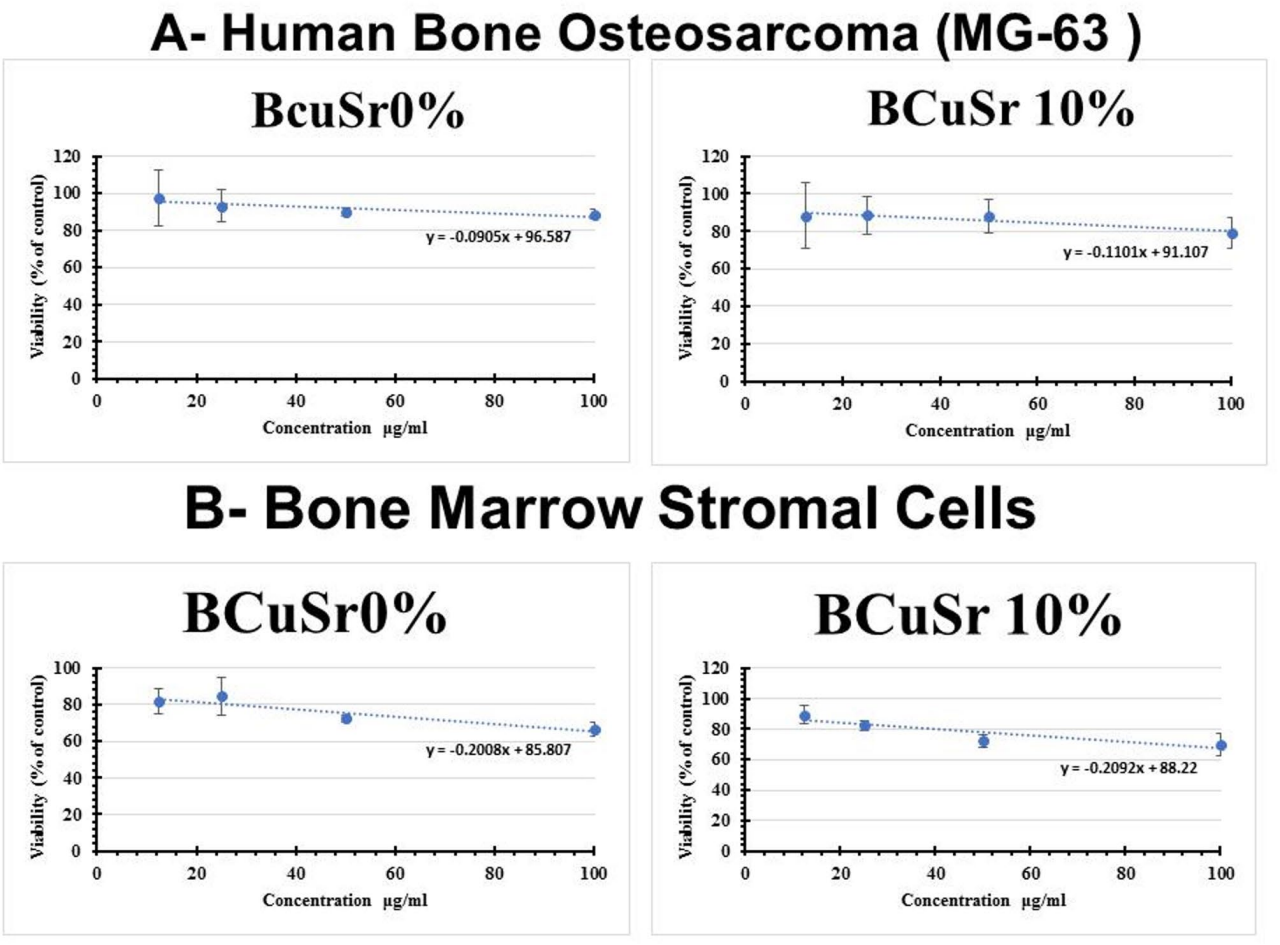
The in vitro testing of the viability cell (**A**) Human bone osteosarcoma of MG-63, (**B**) bone marrow stromal cells using MTT assay test (*p* > 0.05) *n* = 3.

The evaluation of the cytocompatibility of the examined particles was conducted through the MTT assay following their culture with bone marrow stromal cells and the human bone osteosarcoma cell line (MG-63) for duration of 48 h. The decrease in cell viability compared to the control group served as a measure of cytotoxic activity. The findings presented in Fig. 10a indicate that the cell viability of MG-63 remained stable when exposed to the tested particles, achieving values of 88.7 ± 2.59 for BCuSr0% and 79.1 ± 8.3 for BCuSr10% at a concentration of 100 µg/mL, respectively (*p* > 0.05).while in Fig. 10b shows that the cell viability of bone marrow stromal cells is slightly affected by exposure to the tested particles, reaching 66.57 ± 3.5 for BCuSr0% and 69.83 ± 7.64 for BCuSr10% at 100 µg/mL, respectively (*p* > 0.05).While reaching to 72.55 ± 1.8 for BCuSr0% at 50 µg/mL and 82.26 ± 3.3 for BCuSr10% at 25 µg/mL, respectively (*p* > 0.05) The amount of the examined substances needed to eliminate 50% of the cell population (IC50) is calculated using the data collected to create a dose-response curve (Fig. 10). In the human bone osteosarcoma cell line (MG-63), BCuSr 10% showed a slightly elevated IC50 value (182 µg/ml) compared to BCuSr 0% (178 µg/ml) in bone marrow stromal cells, while BCuSr 0% presented a slightly higher IC50 value (514 µg/ml) than BCuSr 10% (373 µg/ml). According to Ali Can Özarslan’s findings, the use of co-incorporated Sr–Cu bioactive biosilica glass-based porous scaffolds improved cell proliferation even at low concentrations. The influence of biosilica, which may contain trace amounts of various elements, may also be regarded as the cause of this increase in cell viability (%) in addition to the action of dual additions on cells^29^. From previous data the results indicated that BCuSr0% is less cytotoxic to bone stromal cells than doped samples at 50 µg while the cell percentage raised to 88.7% and 79.1% for BCuSr0% and BCuSr10% respectively at concentration of 100 µg/ml .All tested that samples showed enhanced cell viability more than 70% was considered safe and nontoxic to cells as previously reported^18^.

## Conclusion

The effective synthesis of nano bioactive glass may be accomplished using a composition of 45 wt% SiO2, 35 wt% CaO, and 20 wt% P2O5 via the rapid alkali-mediated sol-gel method. Both CuO and SrO can function as partial substitutes for CaO in varying quantities. The TEM study verified that all generated samples were at the nanoscale. Diverse characterisation approaches might be utilized to assess the generated materials. The results demonstrated that the incorporation of Cu and Sr ions improves the bioactivity of bioactive glass. Furthermore, it facilitated the synthesis of hydroxyapatite and adjusted the Ca/P ratio nearer to the optimal stoichiometric value of 1.67. Each sample has an exceptional specific surface area; two critical parameters for drug delivery are pore diameter and total pore volume. The research additionally investigated the disintegration and degradability of the glass. Moreover, the produced materials exhibited compatibility with bone marrow cells and effectively treated osteoblast-like cells (MG-63) without causing any morphological alterations. The synthesized nano bioactive glass materials offer a promising pathway for drug delivery applications. The nanoparticles were functionalized with the medication ciprofloxacin to reduce the infection risk linked to orthopedic implantation procedures. The antibacterial efficacy of ciprofloxacin was effectively maintained upon incorporation into nanoparticle matrices. Thus, it may be concluded that the synthesized nanoparticles including ciprofloxacin represent a promising approach for bone regeneration.

## Data Availability

The datasets used and/or analysed during the current study available from the corresponding author on reasonable request.
